# What Can Long Terminal Repeats Tell Us About the Age of LTR Retrotransposons, Gene Conversion and Ectopic Recombination?

**DOI:** 10.3389/fpls.2020.00644

**Published:** 2020-05-20

**Authors:** Pavel Jedlicka, Matej Lexa, Eduard Kejnovsky

**Affiliations:** ^1^Department of Plant Developmental Genetics, Institute of Biophysics of the Czech Academy of Sciences, Brno, Czechia; ^2^Faculty of Informatics, Masaryk University, Brno, Czechia

**Keywords:** transposable elements, LTR retrotransposons, nesting, age estimation, gene conversion, ectopic recombination, plants

## Abstract

LTR retrotransposons constitute a significant part of plant genomes and their evolutionary dynamics play an important role in genome size changes. Current methods of LTR retrotransposon age estimation are based only on LTR (long terminal repeat) divergence. This has prompted us to analyze sequence similarity of LTRs in 25,144 LTR retrotransposons from fifteen plant species as well as formation of solo LTRs. We found that approximately one fourth of nested retrotransposons showed a higher LTR divergence than the pre-existing retrotransposons into which they had been inserted. Moreover, LTR similarity was correlated with LTR length. We propose that gene conversion can contribute to this phenomenon. Gene conversion prediction in LTRs showed potential converted regions in 25% of LTR pairs. Gene conversion was higher in species with smaller genomes while the proportion of solo LTRs did not change with genome size in analyzed species. The negative correlation between the extent of gene conversion and the abundance of solo LTRs suggests interference between gene conversion and ectopic recombination. Since such phenomena limit the traditional methods of LTR retrotransposon age estimation, we recommend an improved approach based on the exclusion of regions affected by gene conversion.

## Introduction

Transposable elements (TEs) are abundant structural and functional genome components inhabiting genomes throughout the course of life evolution. They have evolved into many different types distinguished by structure, mechanisms of spreading and effect on cell functioning. The activity of transposable elements is dependent on the developmental stage, is tissue-specific, epigenetically regulated and often induced by stress. This is evident especially in plants (that are sessile) where TEs represent in large genomes like maize, barley or wheat often more than 85% of the genome (Charles et al., 2008; Schnable et al., 2009; Wicker et al., 2018).

LTR retrotransposons are ancient genome inhabitants present in the genomes of all major taxonomic groups, being abundant especially in plants (Feschotte et al., 2002; Kejnovsky et al., 2012). They exhibit waves of explosive amplification during the evolution of host species that often predate the speciation events (Kim et al., 2004). Since retrotransposon activation is caused by stress (Hirochika, 1997; Grandbastien, 1998), such amplification waves probably corresponded to major environmental challenges such as climate change or pathogen attack. The generation of new retrotransposon copies is balanced by deletions resulting from ectopic recombination and the formation of solo LTRs, leading to genome size either increasing or decreasing (Devos et al., 2002; Ma et al., 2004; Bennetzen et al., 2005; Vitte and Panaud, 2005).

The removal of LTR retrotransposons is caused by two unrelated, ectopic processes: (i) homologous unequal recombination, producing solo LTRs with or without TSDs, intact elements without TSDs and/or recombined elements with LTRs flanked by both PBS and PPT (Shirazu et al., 2000; Vitte and Panaud, 2003), and by (ii) illegitimate recombination, using a mechanism of mis-repair of double strand breaks, as was shown in Arabidopsis (Devos et al., 2002) and wheat (Wicker et al., 2003) and resulting in whole or partial deletion of LTRs (Devos et al., 2002; Ma et al., 2004).

Retrotransposon activity during the course of evolution differs between families and plant species. Some retrotransposon families have short bursts of intense activity for a few 100,000 years while other families have only moderate activity over long periods of time e.g., 1–2 million years (Wicker and Keller, 2007). Such amplifications are more visible in animal genomes (Kim et al., 2004) than in plants because plant genomes are more dynamic and intermingled (Kejnovsky et al., 2009). While in animals endogenous retroviral integrations older than 100 million can be identified (Martins and Villesen, 2011), the high turnover of retrotransposons (birth and decay of elements) in plant genomes prevents the detection of insertions more than tens of million years old (Maumus and Quesneville, 2014). Seminal papers from the beginning of this millenium, analyzing a number of plant species, showed that the majority of LTR retrotransposons were inserted less than three million years ago (Devos et al., 2002; Ma et al., 2004; Bennetzen et al., 2005).

Studies of the evolutionary dynamics of various LTR retrotransposon families are based on the estimation of relative and absolute age (Kijima and Innan, 2009). The age of LTR retrotransposons is mostly estimated using the divergence of 5′ and 3′ LTRs (Gaut et al., 1996; SanMiguel et al., 1998, 2002). However, recent studies have shown that this traditional age estimation method is not absolute, namely because (i) the differences in substitution rates between species (Ma and Bennetzen, 2004) and (ii) the effect of homogenizing processes such as gene conversion (Kijima and Innan, 2009; Cossu et al., 2017).

The absolute age of LTR retrotransposons is calculated using the formula *T* = K/2 × *r*, where *T* = time of divergence, *K* = divergence and *r* = substitution rate (Bowen and McDonald, 2001). However, substitution rates vary between species e.g., 1.6 × 10^–8^ substitutions per site per year in drosophila (Li, 1997), 1.5 × 10^–8^ in Arabidopsis (Koch et al., 2000) and 1.3 × 10^–8^ in grasses (Ma and Bennetzen, 2004). The weakness of the traditional method for LTR retrotransposon and retrovirus integration time estimation using only LTR divergence has been highlighted previously by Martins and Villesen (2011) who developed an improved approach using phylogenetic data. These authors showed that 5′ and 3′ LTR have distinct evolutionary rates.

The need for other approaches for LTR retrotransposon age estimation has led to the development of an alternative method based on the comparison of intra-specific versus interspecific differences in repeats (species-specific elements are younger than conservative elements). This method has been used to date a variety of repeats (not only LTR retrotransposons) in Arabidopsis (Maumus and Quesneville, 2014) and the legume tribe Fabeae (Macas et al., 2015).

Here we measured the LTR divergence of thousands of LTR retrotransposons coming from fifteen plant species to determine their age and thus study their evolutionary dynamics. We found that LTR divergence depends not only on the element age but also on e.g., LTR length. We propose gene conversion as the process complicating age estimation from LTR similarity. In addition, we measured the extent of gene conversion in LTRs as well as its relation to other processes such as solo LTR formation by ectopic recombination.

## Materials and Methods

### Genomic Sequence Sources and TE Annotation

Plant genomes covering diverse taxons of higher plants were downloaded from Phytozome 12.0 (Goodstein et al., 2012). The fifteen species included *Arabidopsis thaliana* (Lamesch et al., 2012), *Arabidopsis lyrata* (Rawat et al., 2015), *Brachypodium distachyon* (International Brachypodium Initiative, 2010), *Chlamydomonas reinhardtii* (Merchant et al., 2007), *Glycine max* (Schmutz et al., 2010), *Lotus japonicus* (Sato et al., 2008)^1^, *Medicago truncatula* (Tang et al., 2014), *Musa acuminata* (D’Hont et al., 2012), *Oryza sativa* (Ouyang et al., 2007), *Physcomitrella patens* (Lang et al., 2018), *Populus trichocarpa* (Tuskan et al., 2006), *Selaginella moellendorffii* (Banks et al., 2011), *Sorghum bicolor* (McCormick et al., 2017), *Solanum lycopersicum* (Tomato Genome Consortium, 2012), and *Solanum tuberosum* (Sharma et al., 2013). The complete workflow of our analysis is visualized as a step-by-step flowchart in Supplementary Figure S1. Unmasked sequences were analyzed with TE-greedy-nester (Lexa et al., 2018). TE-greedy-nester in its latest version relies upon LTR Finder (Xu and Wang, 2007) to identify full-length LTR retroelements. It recursively removes the identified elements from the analyzed genomes so that other full-length copies fragmented by nesting can be identified with the same tools. The annotations were saved as GFF3 files for visualization and downstream analysis. They contained information on the positions of entire elements as well as their structural components [LTR, PBS, PPT, *gag* and *pol* gene protein domain sequences, target site duplications (TSD)]. Subsequences of interest (LTR, RT domain) were extracted from downloaded genome sequences using the bedtools package (Quinlan and Hall, 2010).

The elements, retrieved by TE-greedy-nester, which contain detected LTR retroelement protein domains are also automatically annotated using recent classification by Neumann et al. (2019). The annotation process is based on homology (BLASTX; Altschul et al., 1990) with a custom database consisting of a combination of Cores Seq. from Gypsy Database (Llorens et al., 2011) and polyprotein sequences recently present by Neumann et al. (2019). Therefore, GFF3 outputs were filtered for the presence of at least one protein domain. Further, based on the mutual position of annotated LTR retrotransposons within the genomic sequence the TEs with boundaries present within the coordinates of another TE were simply considered as “nested” and “original,” respectively. *Vice versa*, the solitary TE was labeled as “non-nested.” Finally, in order to minimize the amount of false positive elements detected by TE-greedy-nester, all the non-nested and original elements were filtered for the presence of TSD. The respective counts of LTR retrotransposons used in this study are given in Table 1 and corresponding GFF files of filtered retroelements are provided in the Supplementary Material. The plant species presented in table and all figures are ordered by their genome size in Table 1 and by their taxonomic affiliation in Figures. LTR retrotransposon families labels in Supplementary Figure S3 are presented as a combination of superfamily (i.e., Ty1/Copia and Ty3/Gypsy as “copia” and “gypsy,” respectively) and given families concatenated by underscores (e.g., “gypsy_Athila”).

**TABLE 1:** Summary table of LTR retrotransposon counts and mean age obtained from fifteen plant species.

Species	Label	Class	Family	LTR retrotransposons				
				Genome size [Mbp]	Nested and Original	Non-nested	Sum	Mean age [± SD]
*Glycine max*	Gmax	Eudicots	Fabaceae	978.5	789	2876	3665	1.33 ± 1.28
*Solanum lycopersicum*	Slyc	Eudicots	Solanaceae	823.9	300	1436	1736	2.26 ± 1.65
*Solanum tuberosum*	Stub	Eudicots	Solanaceae	773.0	253	1140	1393	2.17 ± 1.6
*Sorghum bicolor*	Sbic	Monocots	Poaceae	732.2	2881	4591	7472	0.89 ± 0.95
*Physcomitrella patens*	Ppat	Bryopsida	Funariaceae	473.2	633	2478	3111	1.15 ± 1.13
*Lotus japonicus*	Ljap	Eudicots	Fabaceae	462.5	96	656	752	0.63 ± 1.04
*Populus trichocarpa*	Ptri	Eudicots	Salicaceae	422.9	88	726	814	1.19 ± 1.39
*Medicago truncatula*	Mtru	Eudicots	Fabaceae	411.8	139	330	469	2.62 ± 1.77
*Musa acuminata*	Macu	Monocots	Musaceae	390.6	66	572	638	0.56 ± 0.97
*Oryza sativa japonica*	Osat	Monocots	Poaceae	374.5	661	1750	2411	0.9 ± 1.07
*Brachypodium distachyon*	Bdis	Monocots	Poaceae	271.2	137	608	745	1.83 ± 1.23
*Selaginella moellendorffii*	Smoe	Isoetopsida	Selaginellaceae	212.7	111	648	759	1.58 ± 1.45
*Arabidopsis lyrata*	Alyr	Eudicots	Brassicaceae	206.7	155	837	992	0.58 ± 0.8
*Arabidopsis thaliana*	Atha	Eudicots	Brassicaceae	119.1	32	130	162	1.21 ± 1.09
*Chlamydomonas reinhardtii*	Crei	Chlorophyceae	Chlamydomonadaceae	107.1	0	25	25	0.25 ± 0.89
Total					6341	18,803	25,144	

*Average time from their insertion is indicated (million years ago ± Standard deviation).*

### LTR Divergence

The LTR divergence of elements in individual families was obtained from global alignment by STRETCHER tool (Emboss 6.6.0; Rice et al., 2000), expressed as percentage of identical bases in the alignment (LTR similarity). These values served for visualization of LTR similarity and length relationship and subtraction of LTR similarity within each pair of nested and original (pre-existing) element (“delta LTR similarity”). Furthermore, in order to exclude the possibility that the observed negative delta LTR similarity was simply a result of random mutations, we simulated a pair of LTRs subject to mutations with BBMap mutate.sh^2^ and subsequently generated 1000 independent mutations of that pair. For each pair of sequences we calculated the similarity of their global alignment and plotted the distribution of these values as simulated delta LTR similarity.

### Insertion Time Estimation

The nucleotide divergence between aligned sequences (CLUSTALW tool with -output = PHYLIP command; Larkin et al., 2007) was calculated using PhyML (Guindon and Gascuel, 2003) with substitution model K80. Subsequent steps were adopted from Pereira (2004). In order to minimize errors from poor quality alignments retrieved by CLUSTALW, alignments shorter than eighty nucleotides and LTR pairs with divergence (K) value greater than 0.2 were discarded (207 out of 25,144; i.e., less than 1%). Subsequently insertion time was estimated using the formula *T* = *K* / 2*r*, with substitution rate of 1.3 × 10^–8^ per site per year (Bowen and McDonald, 2001; Ma and Bennetzen, 2004).

### Solo LTR Detection

In addition to the GFFs files with information on full-length LTR retrotransposons, the TE-greedy-nester also retrieves respective chromosome sequence remainder after full-length elements removal in FASTA format. These sequences were used for solo LTRs detection, which was conducted in two subsequent steps: (i) LTR_retriever was employed to process split sequences using the default arguments setup (Ou and Jiang, 2018); and (ii) obtained outputs were passed to REannotate software (Pereira, 2008), which clearly distinguish solo LTRs from truncated retroelements containing also uncoupled LTRs or their remnants.

### Determination of Gene Conversion in LTRs and Removal of Converted Parts

In order to estimate the extent of potential gene conversion along the long terminal repeats of LTR retrotransposons we employed GENECONV (Sawyer, 1999) which was shown to be precise and reliable compared with other software (Mansai and Innan, 2010). Moreover this tool has already been used for this specific task in plant LTR retrotransposons (Cossu et al., 2017). GENECONV uses permutation analysis of sequence alignment to determine a probability that two LTR subregions have a common origin due to gene conversion. This is based on the density of nucleotide substitutions in these regions, compared to the background in other parts of the input sequences. Consequently, we are aware that alongside gene conversion, the sequence identities retrieved by GENECONV could be caused also by random processes (for instance, a low overall rate of mutation or multiple testing). We consider our results as “upper limits” and interpret the results as “possible gene conversion” on that account. The LTR pair sequences of all elements from each specific family and plant species were collected within one fasta file. Then all possible pairs of LTRs from two different elements were generated (i.e., 5′ and 3′ LTRs from two elements – four sequences per one fasta file). Thereafter each set of LTRs was aligned using CLUSTALW (Larkin et al., 2007) and subjected to GENECONV using parameters: /w123 /lp /f /eb /g1 -nolog. Because of the extraordinary number of pairs (over 100,000 files) generated in some overrepresented retrotransposon families, the GENECONV run was stopped when LTRs of each element were analyzed with those of at least ten other elements. Pairwise inner fragments from GENECONV output were evaluated and filtered. The first filter was conducted in order to avoid getting false positive results due to multiple comparisons of all possible sequences. Thus the *p*-value retrieved by GENECONV was multiplied by the number of all sequences in the original plant- and LTR retrotransposon family specific multifasta file, and only records with *p*-value < 0.05 were accepted for following steps. Another filter was used in cases where gene conversion fragments overlapped each other and the best candidate was chosen based on the lowest *p*-value and number of mismatches. Further, since the minimal length of gene conversion fragments varied among different organisms (Mansai et al., 2011), we set this value to 50 bp to avoid overestimation of our findings. Finally, for the determination of LTR similarity of original elements prior to gene conversion, the maximal length of a converted fragment was limited up to 80% of given LTR length. The converted part was then clipped, flanking parts joined and LTR similarity determined using global alignment by STRETCHER.

### Effect of Whole Genome Mutation on LTR Similarity – *In silico* Simulation

Changes in the similarities of LTRs with different lengths were additionally analyzed by the following simulation. We took LTRs of different lengths deposited in the Gypsy database^3^ (Llorens et al., 2011) and each LTR sequence was duplicated, the space between two LTRs filled by random sequence with length nine times longer than two respective LTRs (since LTRs constitute 10% of full-length LTR retrotransposon in average). This pseudoelement was then randomly inserted into a generated DNA sequence (1 Mbp long) which represented an artificial genome. Such a genome was subjected to mutation at level ranging from 0.7 to 1.0 (with step of 0.01) using BBMap mutate.sh. The similarity of LTRs were counted by emboss stretcher and plotted against LTR lengths. Because outcomes of all the mutation levels revealed the same pattern, only results at mutation level 0.99 were used for our visualization (Supplementary Figure S5).

## Results

### Evolutionary Dynamics of LTR Retrotransposons in Plants

**FIGURE 1:**
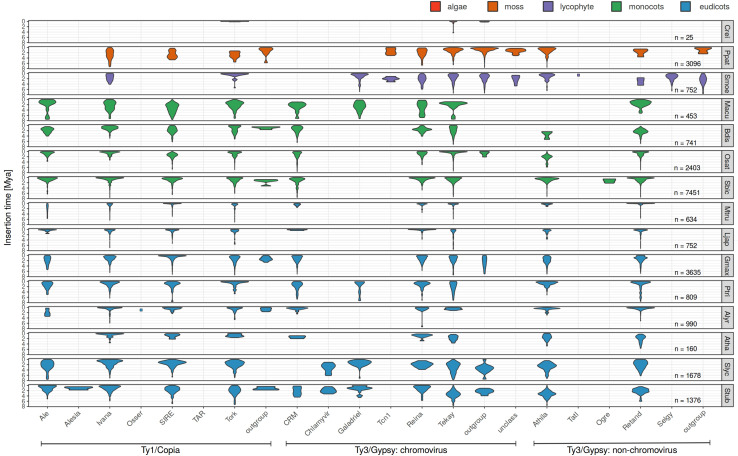
Evolutionary dynamics of LTR retrotransposons of fifteen plant species using LTR divergence method for LTR retrotransposon age estimation. For each species and each family, we measured the abundance of elements having specific LTR divergence to reveal evolutionary expansions and contractions occurring within each family. Nested, original and non-nested LTR retrotransposons were analyzed together.

The LTR similarity in individual families of 25,144 LTR retrotransposons in fifteen plant species (Table 1) was measured and their age determined using the above mentioned formula and substitution rate of 1.3 × 10^–8^ per site per year (Figure 1 and Supplementary Figure S2). This constant was estimated and until now is widely used in grasses (Bowen and McDonald, 2001; Ma and Bennetzen, 2004; Choulet et al., 2010; Zhang and Gao, 2017). In addition, this rate was employed also in Solanum (Xu and Du, 2014) and is close to that established for *A. thaliana* (1.5 × 10^–8^). The overall average insertion time ranges from 0.25 to 2.62 Mya in green alga *Chlamydomonas reinhardtii* and barrel clover *Medicago truncatula*, respectively (Table 1 and Supplementary Figure S2). In Figure 1 LTR retrotransposons were sorted according to a recent LTR retrotransposon classification (Neumann et al., 2019) and plant species were sorted according to phylogeny. The patterns of family expansions differed between retrotransposons as well as between plant species. The age distribution of LTR retrotransposons persisting in one plant species often had similar patterns, despite some visible differences. On the other hand, the evolutionarily dynamics of the same LTR retrotransposon family varied in a number of plant species - some families showed short recent expansion in one species while in another species it had continual moderate activity (Figure 1). Specifically, in rice most of the dominant retroelement families showed recent insertions (Ty1/Copia: Ale, Ivan, Tork; Ty3/Gypsy: CRM, Reina, Retand and Tekay), with the exception of Ty1/Copia SIRE and Ty3/Gypsy Athila (Figure 1). Similarly, in *Sorghum bicolor* all the abundant families were inserted recently. Contrastingly, in the tomato, potato and soybean we found earlier insertions of most LTR retrotransposons families.

**FIGURE 2:**
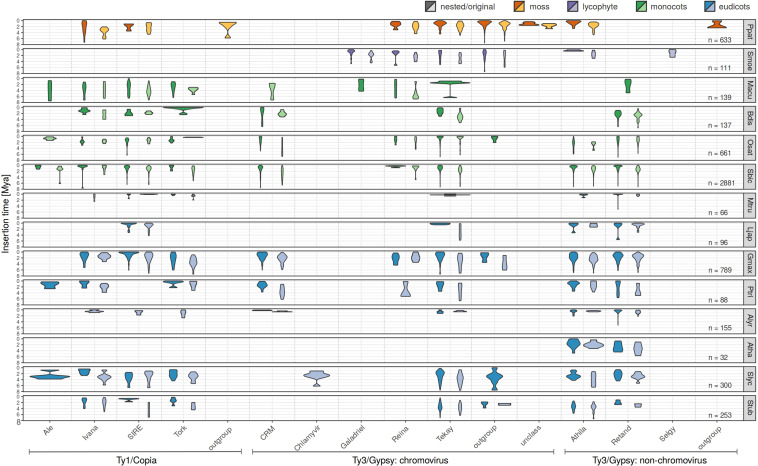
Evolutionary dynamics of nested and original LTR retrotransposons of studied plant species using complex approach for LTR retrotransposon age estimation. For each species and each family, we measured the abundance of elements having specific LTR divergence to reveal evolutionary expansions and contractions occurring within each family. Nested (dark colors), original (light colors) LTR retrotransposons were analyzed separately. Low abundant families were excluded from this visualization.

Separate visualization of nested and original (pre-existing) LTR retrotransposons (Figure 2) showed that (i) nested retrotransposons are, as expected, mostly younger compared to the original ones (see e.g., SanMiguel et al., 1998 for comparison) and (ii) nested elements showed recent expansion in many families.

**FIGURE 3:**
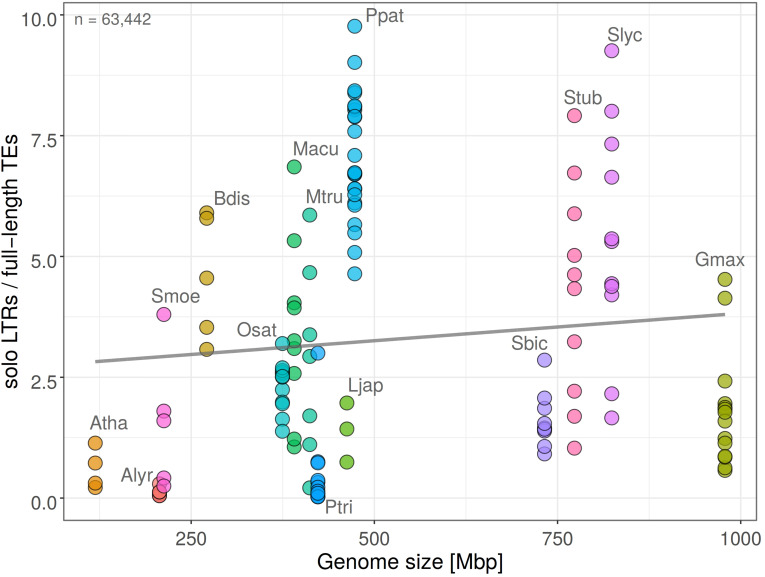
Ratio of solo LTR to full-length retrotransposons (solo LTR/FL) plotted against genome size in fifteen plant species. Each filled circle corresponds to one chromosome, plant species are labeled by different colors (*y* = 2.69 + 0.00114*x*; *R*^2^ = 0.011; Pearson’s *r* = 0.1038 with *p*-value = 0.2363). Total count of solo LTRs is indicated. Higher solo LTR/FL ratio is observed in larger genomes and corresponds to LTR retrotransposon removal by ectopic recombination.

Ectopic (unequal) recombination contributes, together with illegitimate recombination, to element removal and genome contraction. In order to detail how this process is related to the expansion of individual retrotransposon families we measured the ratio of solo LTR to full length elements (solo LTR/FL). We found that in the analyzed species the ratio of solo LTR/FL did not change significantly in dependence on genome size (Pearson’s *r* = 0.1038 with *p*-value = 0.2363; Figure 3), indicating the similar removal of an LTR retrotransposon by ectopic recombination in large and small genomes. This trend was observed in a wide range of species. The proportion of solo LTR significantly differed between individual chromosomes of the same plant species (Figure 3).

### LTRs of Nested Elements Are Often More Diverged Than Original (Pre-existing) Elements

To assess the factors contributing to the similarity of 5′ and 3′ LTRs of the same retrotransposon we compared LTRs in 4126 pairs of nested and original (pre-existing) LTR retrotransposons. Nesting is an absolute measure of relative age – the nested element is always younger than the original and thus the similarity of the nested (younger) element should always be higher than the original (older) element. We named the difference of LTR similarity of nested and original elements as “delta LTR similarity” and expected it to always be positive. Negative delta LTR similarity can be a result of processes that affect the LTRs after insertion, such as the homology-driven form of recombination reshaping LTRs - gene conversion. By filtering the original LTR retrotransposons for the presence of TSDs we minimized the possibility of improper element delineation by TE-greedy-nester.

**FIGURE 4:**
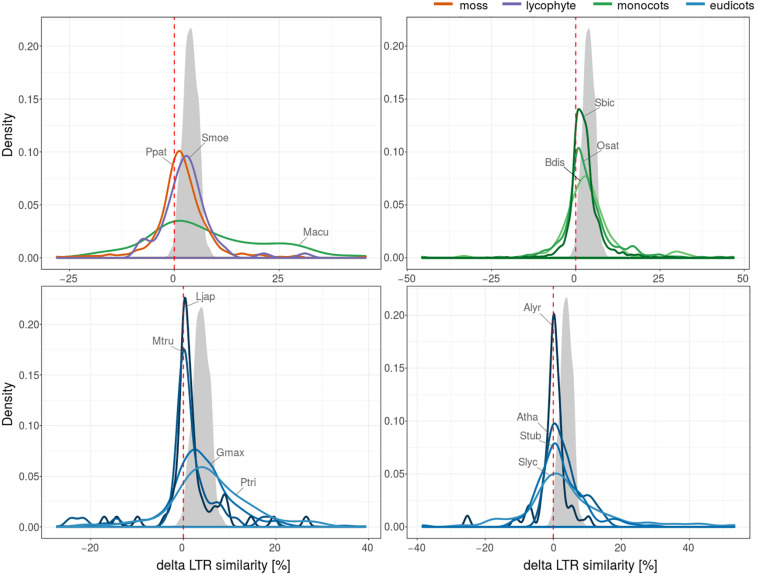
5′-3′ LTR similarity in nested and original LTR retrotransposons. Plant species were divided into four subfigures for better readability. Plotted values represent probability density function based on kernel density estimation. Number of LTR retrotransposons with values of delta LTR similarity (positive values correspond to higher LTR similarity of nested than original elements) for LTR retrotransposons in studied plant species (4126 nested/original pairs). The gray area shows simulated delta LTR similarity distribution under the assumption that only randomly distributed point mutations affected a pair of nested LTRs inserted into another pair with 97% LTR similarity at the time of insertion. The simulated nested structure was then further mutated with mutate.sh at 10% of positions, on average and delta LTR similarity was calculated.

We performed this analysis on fifteen plant species and, surprisingly, we found that the delta LTR similarity was often negative i.e., the similarity of nested elements was lower compared to the similarity of original elements (Figure 4). The proportion of pairs with negative delta LTR similarity (higher similarity of original than nested elements) was 25% (1042 of 4126) and varied in individual species (Figure 4). To rule out the possibility that the observed negative results were simply due to random mutations, we simulated a pair of LTRs with BBMap mutate.sh^4^ generating 1000 independent mutations. For each pair of sequences we calculated the similarity of their global alignment and plotted the distribution of these values as simulated delta LTR similarity (gray area, Figure 4).

### Longer LTRs Have a Higher 5′-3′ LTR Similarity Than Shorter Ones

**FIGURE 5:**
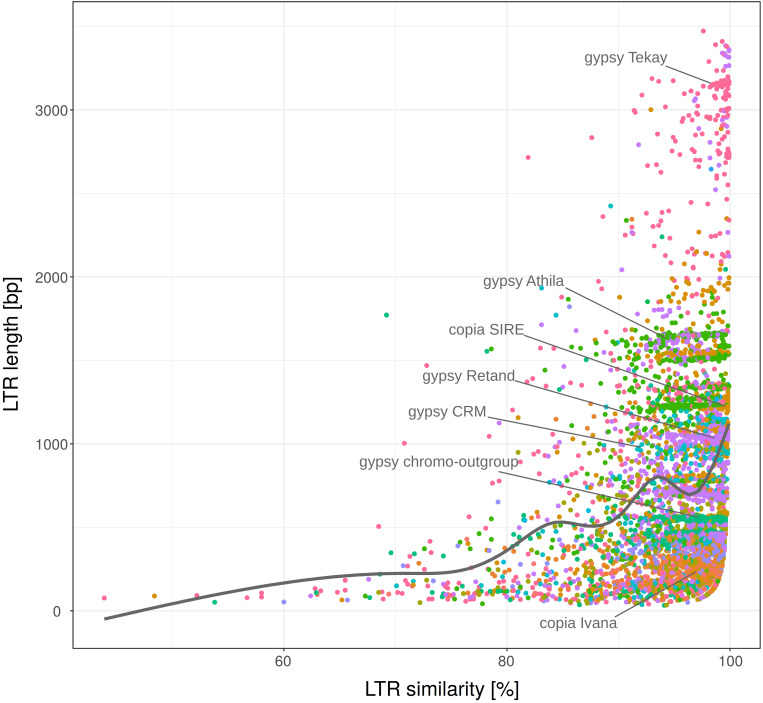
LTR length plotted against 5′-3′ LTR similarity. LTR retrotransposon families (labeled with different colors) of fifteen plant species. Nested, original and non-nested LTR retrotransposons were analyzed together. Full set of 25,144 elements was randomly sampled to subset with *n* = 5000. Most abundant families are labeled within the plot.

The age of LTR retrotransposons is mostly determined by a traditional method measuring LTR similarity, based on the fact that the 5′ and 3′ LTRs are identical at the time of insertion and accumulate mutations and diverge as an element gets older. However, during our analyses we found that LTR similarity surprisingly positively correlated with the LTR length (Figure 5). The LTRs longer than the median (552 bp) comprised 57 and 65% of the LTRs with 95 and 99% similarity, respectively. This suggests that factors other than age have contributed to the similarity of the LTRs. LTR length density of the most abundant retrotransposon families (Ty1/copia: Ivana, SIRE and Tork; Ty3/gypsy: Athila, CRM, Reina, Retand and Tekay) culminated twice, around 300 and 1000 bp (Supplementary Figure S3).

### The Extent of Gene Conversion

**FIGURE 6:**
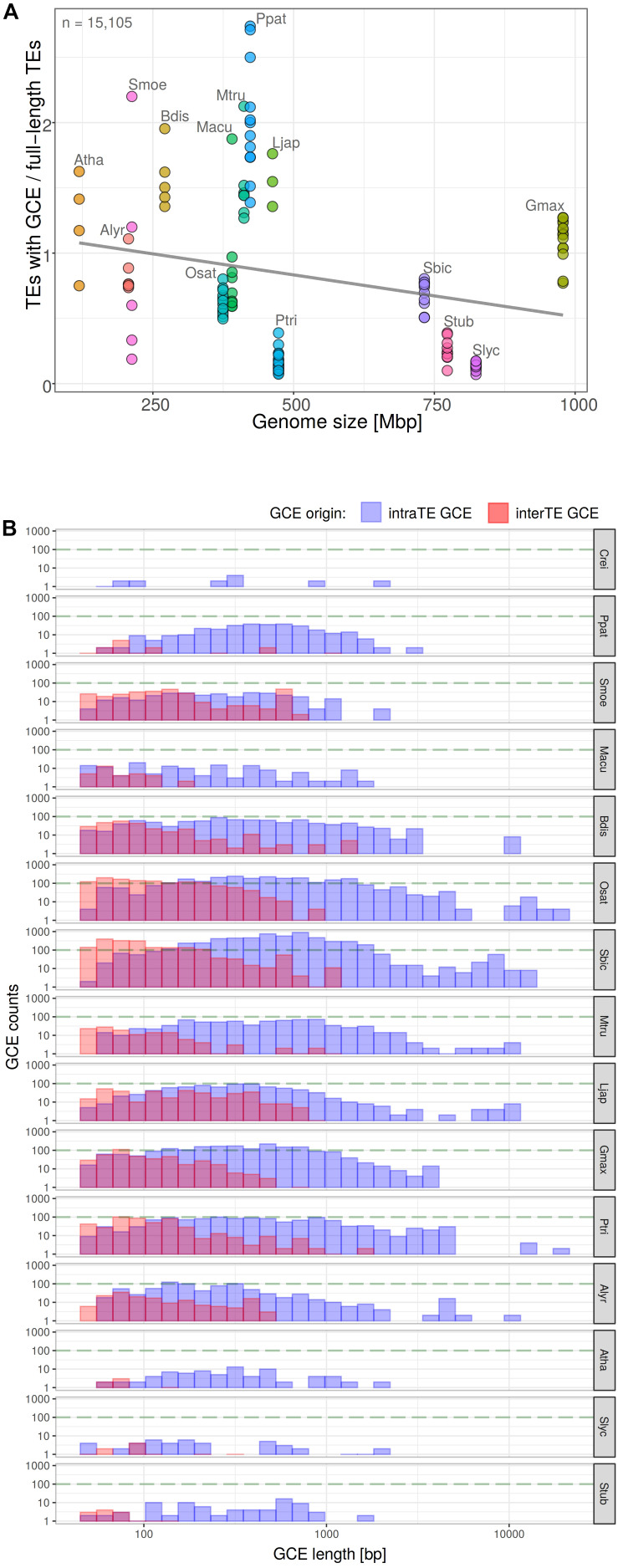
Measurement of gene conversion events (GCE) along the LTR retrotransposons by GENECONV software. Proportion of LTR retrotransposons with GCE plotted against genome size in plant genomes **(A)** (*y* = 1.16 – 0.000644*x*; *R*^2^ = 0.059; Pearson’s *r* = –0.2420 with *p*-value = 0.005175). Total count of elements with GCE is indicated. Each filled circle corresponds to one chromosome, plant species are labeled by different colors. The GCE length distribution with respect to the origin of GCE donor LTR i.e., from the same element or from the other one **(B)**.

In order to find a possible explanation for the anomalies described above, we analyzed the extent of potential gene conversion along the long terminal repeats of LTR retrotransposons using GENECONV software. Pairwise inner fragments from GENECONV output were evaluated and filtered for gene conversion length and overlaps, e-value and number of mismatches (see section “Materials and Methods”). After quality filtering we calculated (i) the number of LTR retrotransposon containing gene converted regions in dependence on genome size of host species (Figure 6A) and measured (ii) the length of converted regions (Figure 6B). Both the number of elements with converted regions and the length of converted region differed among plant species. Gene conversion negatively correlated with genome size (Pearson’s *r* = −0.2420 with *p*-value = 0.005175; Figure 6A). The length of converted regions (i) varied most often between 100 and 1000 bp and (ii) was higher in the case of gene conversion between LTRs of the same element (intra-element conversion) than for conversion between LTRs of different elements (inter-element conversion). The highest lengths of converted regions were found in *O. sativa*, *P. trichocarpa*, and *S. bicolor* (Figure 6B).

**FIGURE 7:**
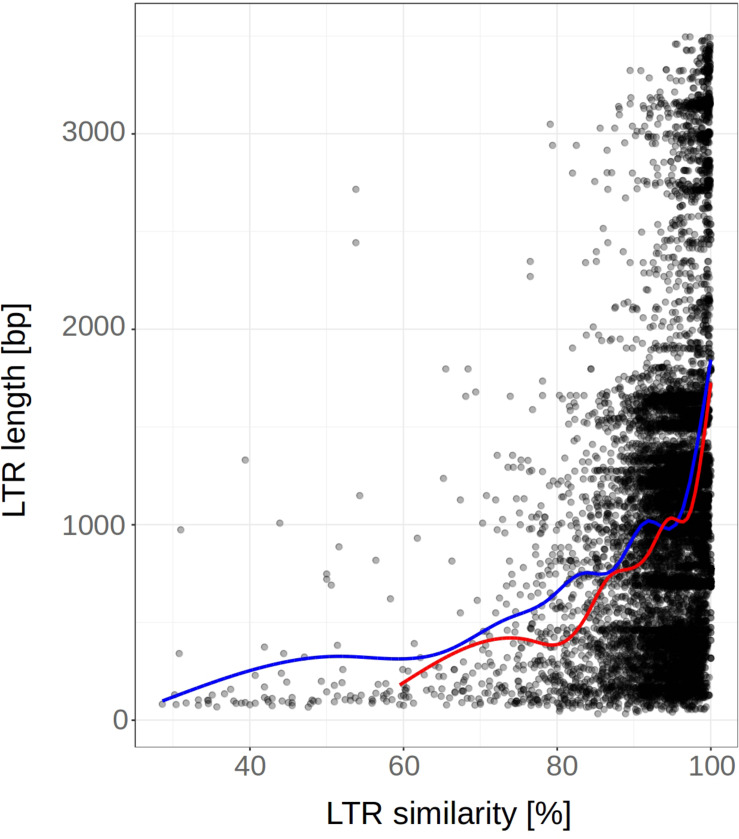
LTR length plotted against the 5′-3′ LTR similarity before and after removal of gene converted regions (predicted by GENECONV). LTR retrotransposon of fifteen plant species (*n* = 5812). Smooth curves (fitted using “loess” method) are plotted for LTRs before and after GCE removal (red and blue color, respectively). Nested, original and non-nested LTR retrotransposons were analyzed together. The removal of converted regions from the LTRs has shifted the curve to the left resulting in an increase of LTR retrotransposon age estimates.

When we removed converted regions (predicted by GENECONV) from the LTRs, we found that the curve showing dependence of LTR similarities on LTR length was shifted to the left. This indicates that LTR similarities have decreased, leading to an increase in the LTR retrotransposon age estimates (Figure 7). When linear trendline was used, the slope after the removal of converted regions decreased (Supplementary Figure S4). However, the strong increase of LTR at the highest LTR similarities was not affected by the removal of converted regions. This possibly suggests that the increase of LTR similarity with length can be caused by other factors or by an unknown technical issue.

In order to better assess the strong increase of LTR similarity in the longer LTRs (even after the removal of converted regions), we performed the following simulation: we took set of LTRs with different length deposited in the Gypsy database (Gydb; *n* = 413), separately inserted the pairs of LTRs (imitating 5′ and 3′ LTR of retrotransposon) into the artificial genomes (always 1 Mb long) and mutated these genomes to a level ranging from 0.7 to 1.0. For each mutation level we found that the distribution of the longer LTRs were always more homogenous than the shorter ones (Supplementary Figure S5 demonstrated mutation level 0.99). Such a finding suggests that this technical phenomenon, in addition to gene conversion, can explain the increase of LTR similarity in longer LTRs as observed in Figure 5.

### The Relationship Between Gene Conversion and Ectopic Recombination

**FIGURE 8:**
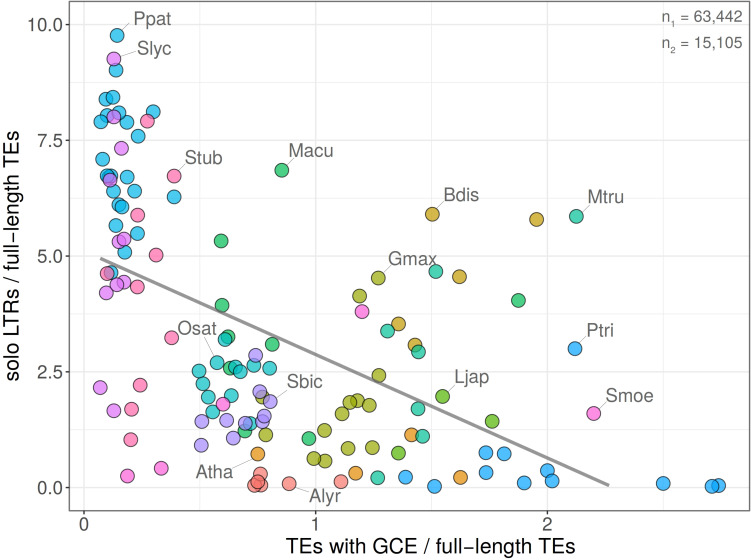
Relationship between gene conversion and solo LTR formation. Dependence of gene conversion events (GCE) predicted by GENECONV plotted against the ratio of solo LTR/FL (*y* = 5.11–2.23*x*; *R*^2^ = 0.29; Pearson’s *r* = –0.5428 with *p*-value = 1.784e-11). Chromosomes with no records of GCE and/or solo LTRs were excluded. Total counts of solo LTRs and elements with GCE are indicated (*n*_1_ and *n*_2,_ respectively). Each filled circle corresponds to one chromosome, plant species are labeled by different colors. The graph shows that chromosomes containing high proportions of gene converted element LTRs contain low proportions of solo LTRs.

Our further analysis was motivated by the speculation that homogenization of retrotransposon families by gene conversion could accelerate ectopic recombination. Such a process would respond to family expansion threatening the host. Therefore, we measured in fifteen plant species the correlation between the intensity of gene conversion predicted by GENECONV and the ratio of solo LTR/FL. We found that the number of LTR retrotransposons exhibiting signs of gene conversion negatively correlated with the proportion of solo LTRs i.e., families exhibiting stronger signs of gene conversion had a lower proportion of solo LTRs (Pearson’s *r* = −0.5428 with *p*-value = 1.784e-11; Figure 8). The remarkable position in the plot showed genomes of *Physcomitrella patens*, *Solanum lycopersicum*, and *S. tuberosum* hosting elements with high values of solo LTR/FL and low proportion of gene conversion (up to 20%). On the other hand, the genome of *Chlamydomonas reinhardtii* contained LTR retrotransposon strongly affected by gene conversion but having very low proportion of solo LTRs. Both extremes support the view that gene conversion and ectopic recombination interfere.

## Discussion

Our results show that (i) evolutionary dynamics of individual LTR retrotransposons differ among retrotransposon families and plant species, (ii) the commonly used LTR retrotransposon age estimation method based on LTR divergence is not absolute, probably due to the influence of gene conversion, (iii) families exhibiting signs of gene conversion less readily form solo LTRs, and (iv) the proportion of solo LTRs did not change with genome size, indicating a similar intensity of ectopic recombination in small and large genomes.

Our LTR retrotransposon age estimates were lower than estimates published by Bennetzen et al. (2005). This difference can be explained by the fact that (i) we used a much higher number of elements (hundreds and thousands compared to tens of elements in most species used by Bennetzen et al., 2005) and (ii) we used constant (1.3 × 10^–8^ in grasses) derived from grasses while Bennetzen et al. (2005) used the constant (6.5 × 10^–9^) originating from maize (SanMiguel et al., 1998).

The age distribution of a range of LTR retrotransposon families in fifteen plant species indicates that retrotransposon activity differed among families, probably as a result of an interplay of various genomic and environmental factors. Such an observation is in accordance with the concept of the genome as an ecosystem of varied elements exhibiting a spectrum of interactions from parasitism via competition to collaboration. Nevertheless, despite the differences in age distribution patterns, some similarities of the expansion profiles in several LTR retrotransposon families of the same species were evident and could reflect stresses that a species underwent when selected retrotransposon families were simultaneously activated.

Some of our results are necessarily affected by technical issues. While we used reasonable settings of TE-greedy-nester and subsequent filtering for minimal full-length TE structure and TSDs as evidence of real insertions, these settings and filtering steps are currently notoriously error-prone and could affect our results. Also, the age estimates (Table 1) could be affected by the quality of genome assembly. Namely, the average age of LTR retrotransposons in Solanum species (tomato and potato plants) was higher compared to other analyzed species here. High number of phylogenetically older retroelements (e.g., Ty3/gypsy: chromo outgroup and Galadriel; Figure 1) was found also in genomes of algae and mosses (Neumann et al., 2019). This putatively false (higher) age determination could be explained by the worse quality of LTR retrotransposon assembly (e.g., when chimeric elements are assembled from different families resulting in their higher distance from the consensus). Our assumption is supported by recent report on lower quality of tomato assembly (Hosmani et al., 2019).

Our finding that LTR similarity depends not only on the retrotransposon age but also on the LTR length (Figure 5) could be partially explained by absence of older longer LTRs, since they are more prone to unequal recombination (Du et al., 2012). The potential involvement of other factors affecting LTR retrotransposon age estimation is also supported by the lower LTR similarity of nested elements compared to the pre-existing ones. Our analysis using GENECONV software predicting the presence of gene conversion indicates that this process is probably responsible for the limitations of the LTR divergence method.

Our results are in accordance with the finding of Cossu et al. (2017) who reported that the length of LTR and the whole LTR retrotransposons (rather than sequence similarity) appears to be a major determinant of the gene conversion frequency. We also showed that gene conversion negatively correlates with the formation of solo LTRs. Compared to Cossu et al. (2017) here we analyzed more plant genomes and more elements and used the whole elements retrieved from the complete genome instead of Illumina reads. The importance of LTR length in an intensity of gene conversion was previously proposed by Du et al. (2012) who showed that the ratio of solo LTR to complete LTR retrotransposons correlates with a number of element features, such as LTR length. The potential role of gene conversion in homogenization of transposable elements was suggested decades ago for yeast Ty elements (Roeder and Fink, 1982), primate SINE elements (Kass et al., 1995), and human Alu elements (Roy et al., 2000). Gene conversion of LTR retrotransposons was proposed to be stronger on non-recombining Y chromosomes than on other chromosomes (Kejnovsky et al., 2007). Gene conversion has also been observed in satellite DNA (Krzywinski et al., 2005) and ribosomal genes (Lim et al., 2000).

The non-allelic gene conversion among long terminal repeats has been studied in human endogenous retroviruses recently (Trombetta et al., 2016). The authors suggest that ectopic recombination among LTRs is rather common and could also take place between elements occupying different chromosomes. Here we show that gene conversion between intra-element LTRs is much more frequent than between LTRs of two different elements in plant LTR retrotransposons.

The negative correlation between gene conversion and solo LTR formation indicates that gene conversion does not accelerate ectopic recombination by homogenizing LTRs of the same elements, as we expected, but rather that both processes (gene conversion and ectopic recombination) probably are influential. Therefore, homologous LTRs susceptible to recombination events, are responsive to either ectopic recombination or gene conversion. Both processes are homology-driven and differ in whether or not they resolve in crossing-over.

The presence of gene conversion has almost certainly led to underestimations of LTR retrotransposon age in many studies using the LTR divergence method. Recently, Maumus and Quesneville (2014) cast doubt on the popular dating approach that only assesses the LTR divergence widely applied in plants and stressed the need to use alternative methods based on e.g., reconstruction of ancestral/consensus repeats established from several related species. These authors evidenced such an approach by providing a higher age estimation of TEs in *A. thaliana* (Maumus and Quesneville, 2014) compared to the LTR divergence method. Similarly, Giordano et al. (2007) recommended the use of the genome-wide defragmentation approach for the estimation of TE age providing chronological order of elements rather than the use of an older method based on divergence from a derived consensus (Jurka, 1998). Retrotransposon age underestimation obtained by the LTR divergence method also agrees with the conclusion that LTR retrotransposons in Drosophila are much younger than the host species in which they reside (Bowen and McDonald, 2001). Taken together, the optimization of methods for LTR retrotransposon age estimation should be a subject of further research.

The extent of gene conversion can be affected not only by the LTR length but also possibly by the distance between LTRs, as was shown for duplicated genes (Ezawa et al., 2006), and especially by epigenetic factors such as e.g., chromatin structure (Cummings et al., 2007). Since reversely transcribed cDNA molecules are often used as templates in gene conversion (Doolittle, 1985; Derr and Strathern, 1993; Benovoy and Drouin, 2009), and RNA molecules participate in gene conversion (Doolittle, 1985; Derr et al., 1991; Derr, 1998), then even transcriptional activity of specific LTR retrotransposon families could contribute to such homogenization. Thus, the expression of genome, induced by environmental or endogenous factors, can change the genome structure by homogenization of repetitive DNA.

The interplay between gene conversion and ectopic recombination can oppose LTR retrotransposon amplifications and lead to genome size reduction. This way, gene conversion can fulfill an important regulatory role in genome repeat expansions and contractions as well as related genome rearrangements. Since the activity of transposable elements is epigenetically regulated (Fedoroff, 2012), both gene conversion and ectopic recombination may respond to environmental challenges and thus contribute to eukaryotic evolvability and a higher genome dynamism in plants (Kejnovsky et al., 2009).

## Conclusion and Perspectives

LTR retrotransposons have colonized plant genomes throughout the whole course of evolution. Estimation of LTR retrotransposon age is thus of great importance for the study of plant genome evolution as well as for understanding transposable element biology. Recent research indicates that the traditional age estimation method based on the LTR divergence has some limits, mostly due to the action of gene conversion. Here, we have extended the available knowledge and showed that (i) LTR similarity depends on LTR length and (ii) nested elements often have lower LTR similarity that pre-existing original ones. We have found regions in LTR with signs of gene conversion responsible for both phenomena. Negative correlation between the extent of gene conversion and the abundance of solo LTRs indicates that gene conversion probably interferes with the ectopic recombination between LTRs. Our findings demonstrate that the LTR divergence method should be used carefully keeping in mind the effect of other factors such as gene conversion. We conclude that more methods should be combined for a more reliable LTR retrotransposon age estimation, using e.g., retrotransposon family variability or mutual nesting of elements in order to achieve absolute chronology.

## Data Availability Statement

Publicly available datasets were analyzed in this study. This data can be found here: Phytozome 12.0 unmasked genomes; https://phytozome.jgi.doe.gov/pz/portal.html.

## Author Contributions

PJ and ML analyzed the data. EK and ML conceived the study. All authors wrote the manuscript, read and approved the final manuscript.

## Conflict of Interest

The authors declare that the research was conducted in the absence of any commercial or financial relationships that could be construed as a potential conflict of interest.

## Footnotes

1 ftp://ftp.kazusa.or.jp/pub/lotus/lotus_r2.5/Lj2.5_genome_contigs.fna.gz

2 https://github.com/BioInfoTools/BBMap

3 http://gydb.org/images/9/94/LTRs_and_TIRs.zip

4 https://github.com/BioInfoTools/BBMap
