# Supplementary material for: What Can Long Terminal Repeats Tell Us About the Age of LTR Retrotransposons, Gene Conversion and Ectopic Recombination?
Source: Front Plant Sci. 2020 May 20;11:644. doi: 10.3389/fpls.2020.00644 (PMC7251063; doi:10.3389/fpls.2020.00644)

Figure S1. Analysis flowchart

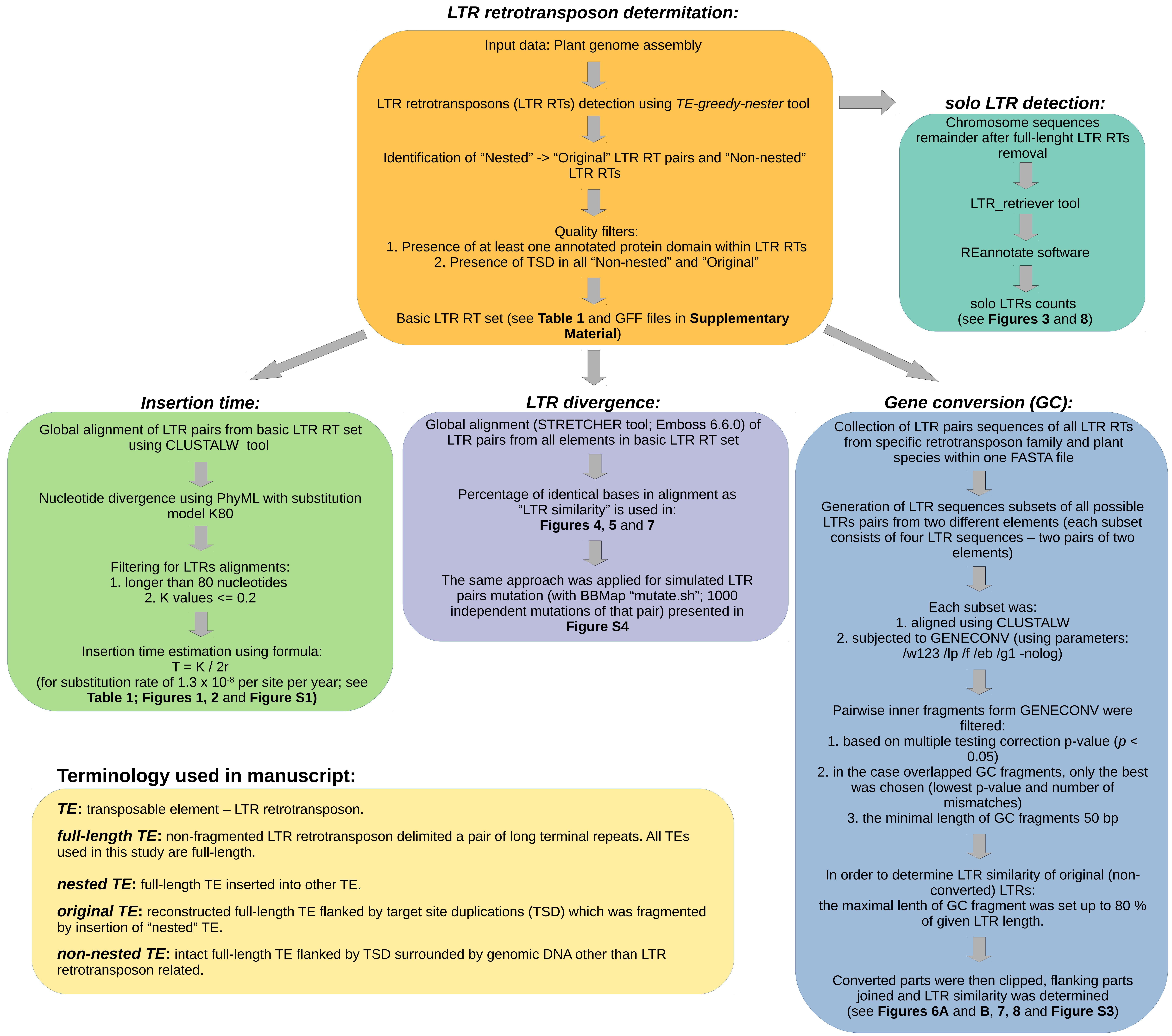

Figure S2. The age distribution of LTR retrotransposons in fifteen plant species. Plotted values represent probability density function based on kernel density estimation. Number of LTR retrotransposons and their average age (± S.D.) are indicated.

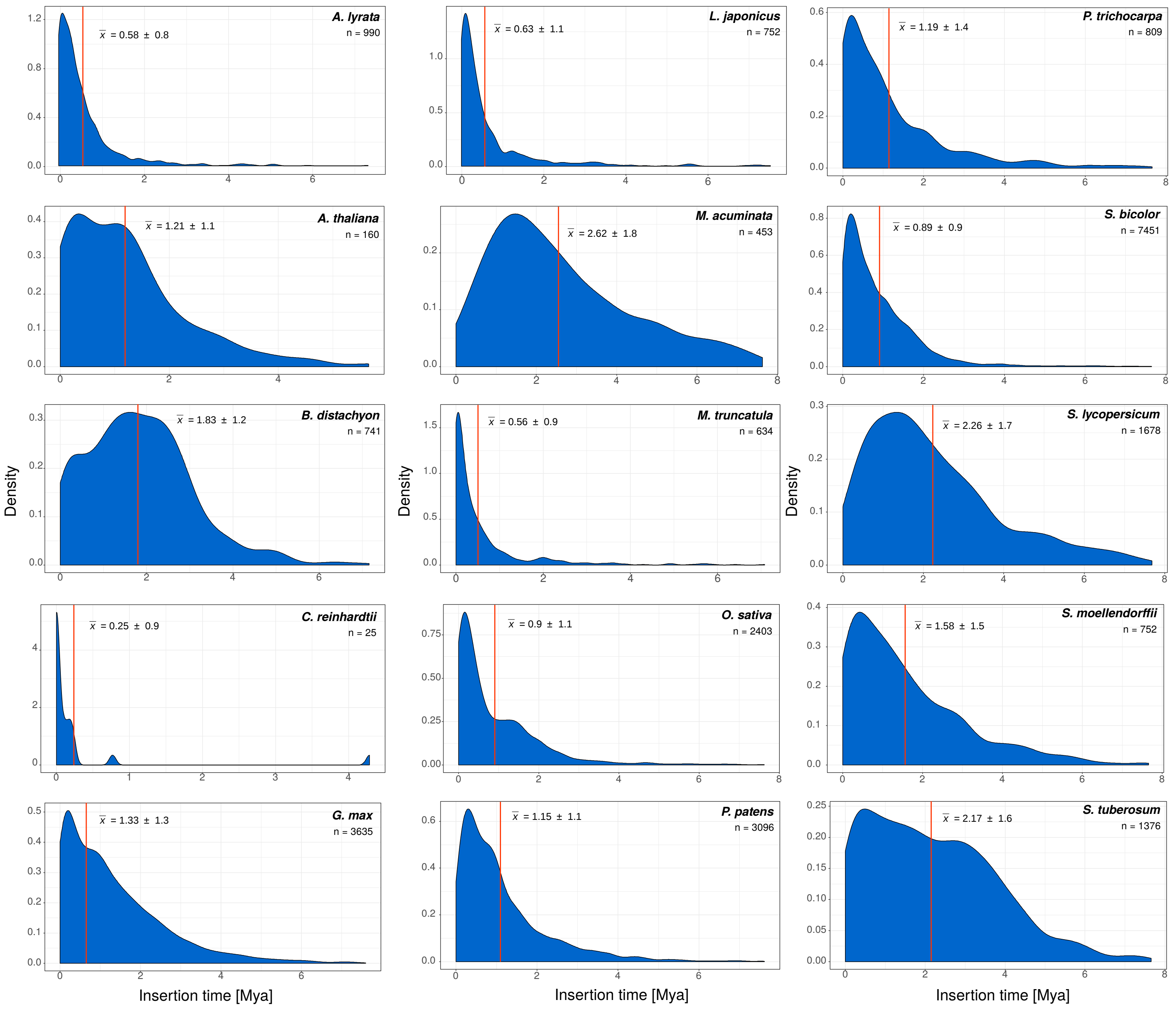

Figure S3. The length of LTRs in individual retrotransposon families.

Low abundant families were excluded from this visualization.

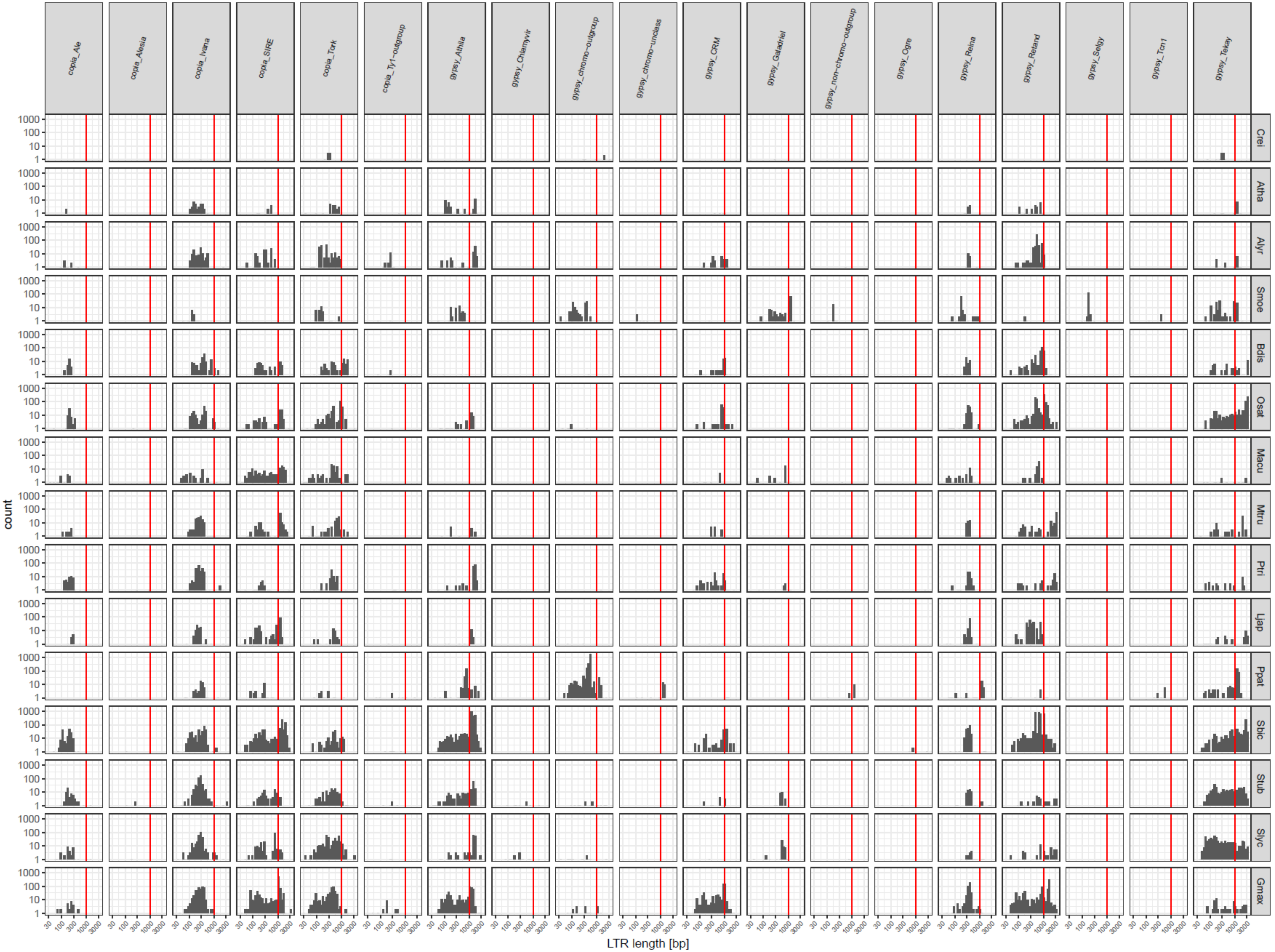

Figure S4. LTR length plotted against the 5'-3' LTR similarity before and after removal of gene converted regions (predicted by GENECONV). LTR retrotransposon of fifteen plant species (n = 5812). Nested, original and non-nested LTR retrotransposons were analyzed together. Linear regression curve with equations and regression coefficients are given.

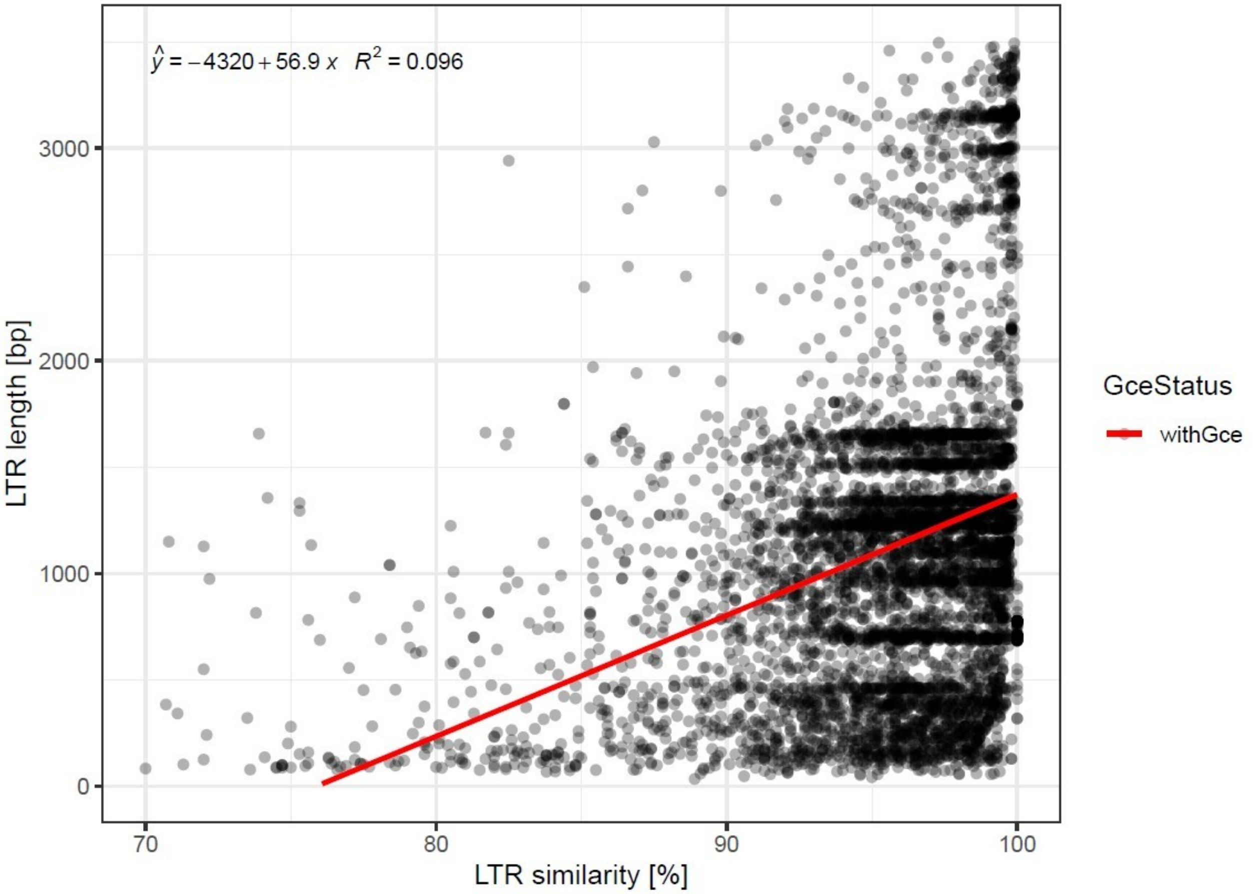

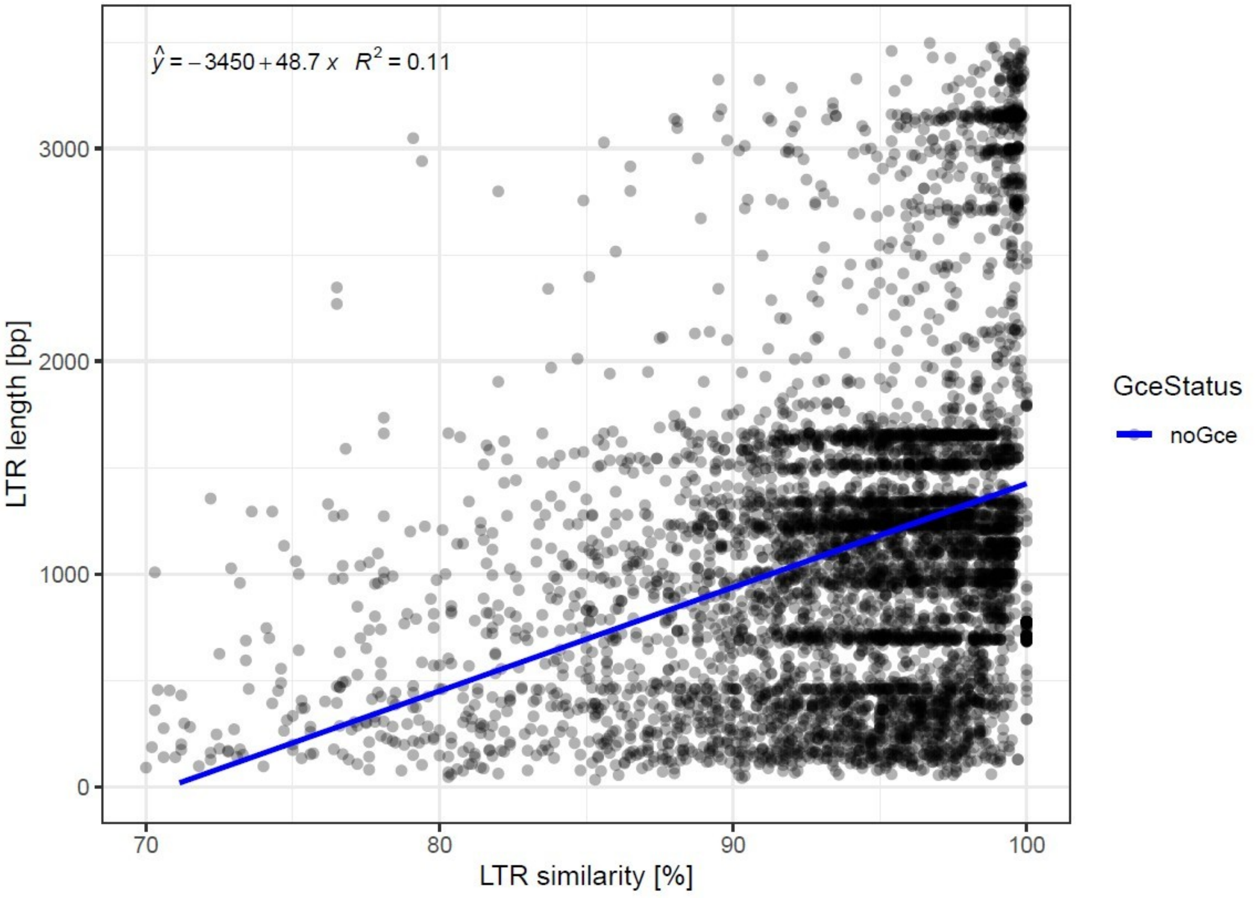

Figure S5. In silico simulation of the effect of LTR mutation on the correlation between LTR length similarity. Horizontal red dashed line denotes median LTR length (398 bp) of analyzed LTRs (n = 413)

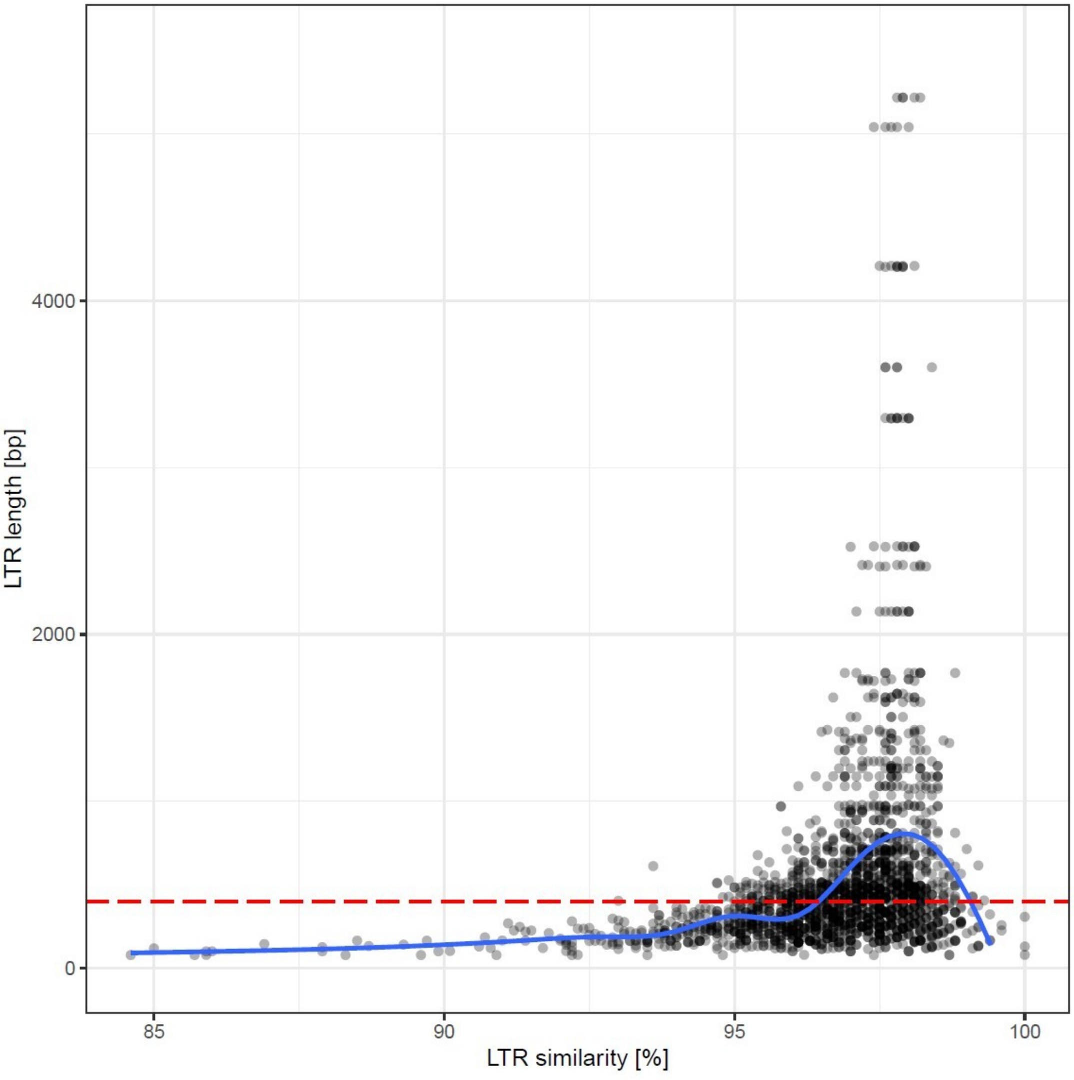

Supplement: Supplementary file 2 [file Data_Sheet_2.PDF]
